# Supplementary material for: Development of an ultrahigh affinity, trimeric ACE2 biologic as a universal SARS-CoV-2 antagonist
Source: Commun Biol. 2025 Oct 6;8:1428. doi: 10.1038/s42003-025-08819-w (PMC12501226; doi:10.1038/s42003-025-08819-w)
Supplement: Supplementary file 2 — Supplementary Information [file 42003_2025_8819_MOESM2_ESM.pdf]

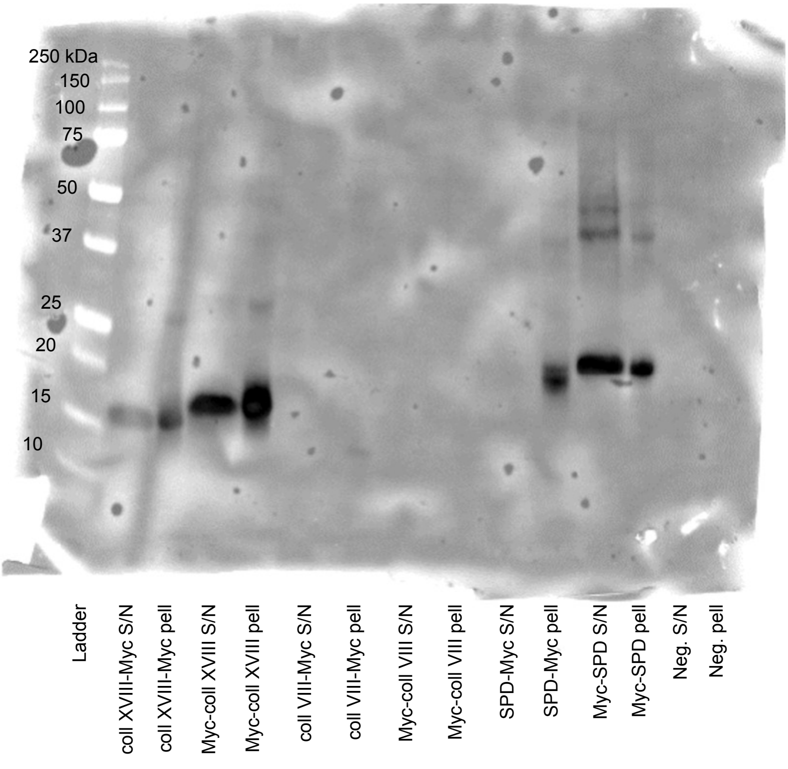

SI Fig. 1: Myc tag expression using various trimerization domains. SDS-PAGE gel (4-20% Mini-PROTEAN stain-free gels, BioRad) transferred to a nitrocellose membrane, blocked with 2% dry milk in PBS, and probed with 1:500 dilution HRP-conjugated anti-Myc antibody, *n* = 1.

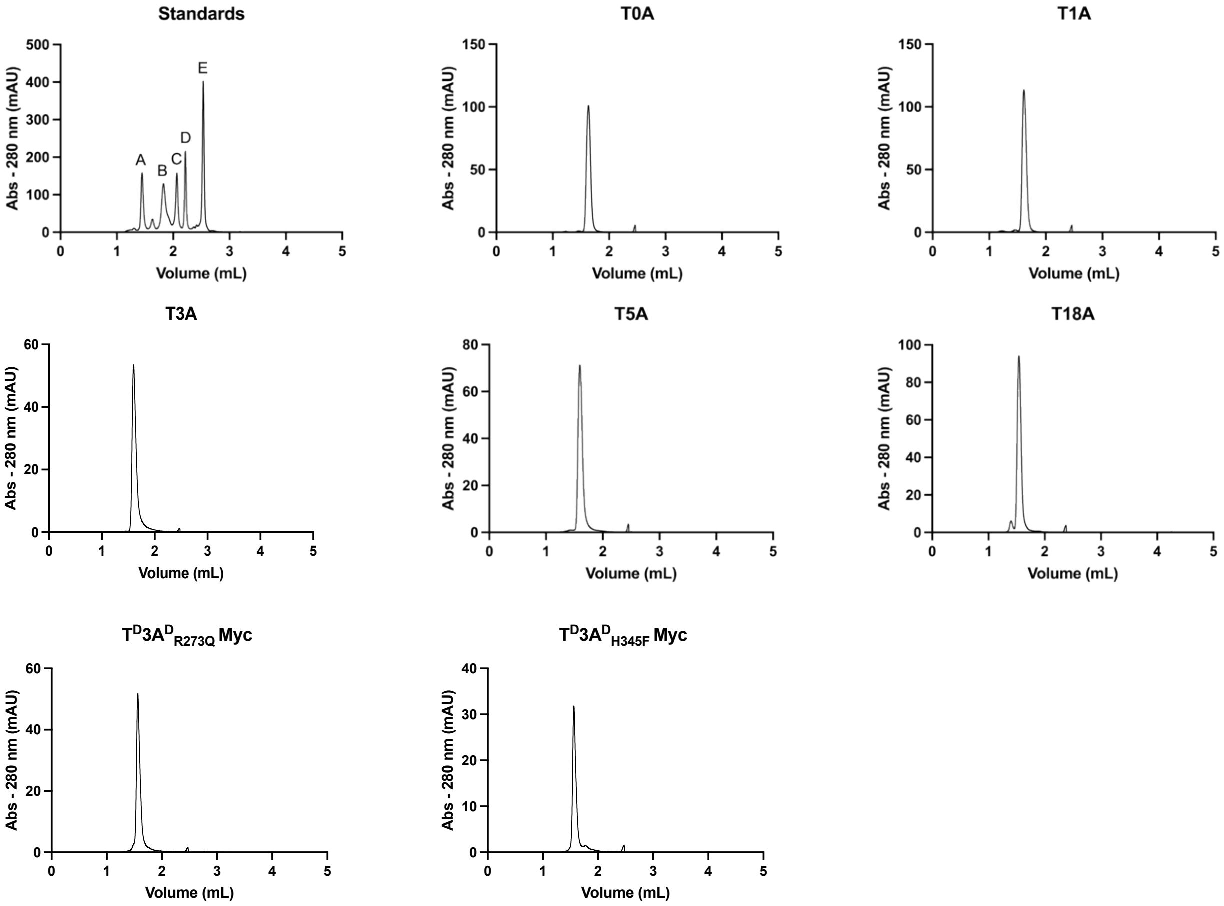

SI Fig. 2: Size exclusion chromatographs to assess purity and stability of ACE2 constructs. High-performance liquid chromatography-size exclusion chromatography (HPLC-SEC) was used to assess purity and stability of all ACE2 constructs.

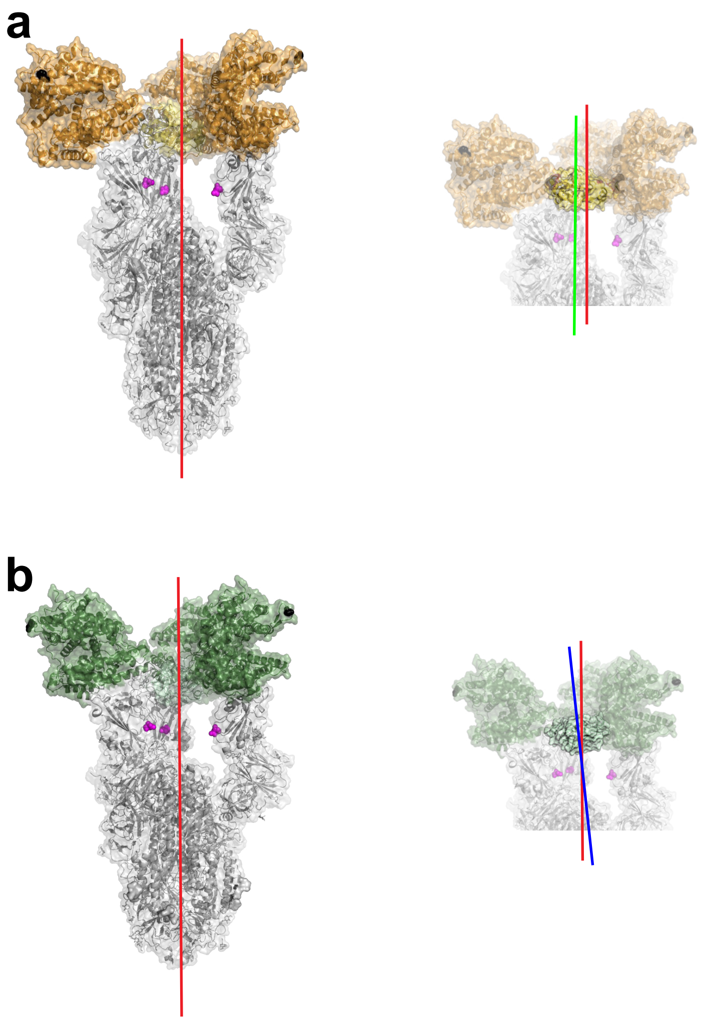

SI Fig. 3: Symmetry of ACE2 trimer binding spike HexaPro protein. Line of symmetry (C3) drawn through modelled SARS-CoV-2 spike HexaPro protein (red arrows) and collagen XVIII domain of for (a) T0A (green arrow), and (b) T3A (blue arrow), respectively. Residue D428 of spike protein shown in magenta, for reference. Lines of symmetry generated using PyMOL.

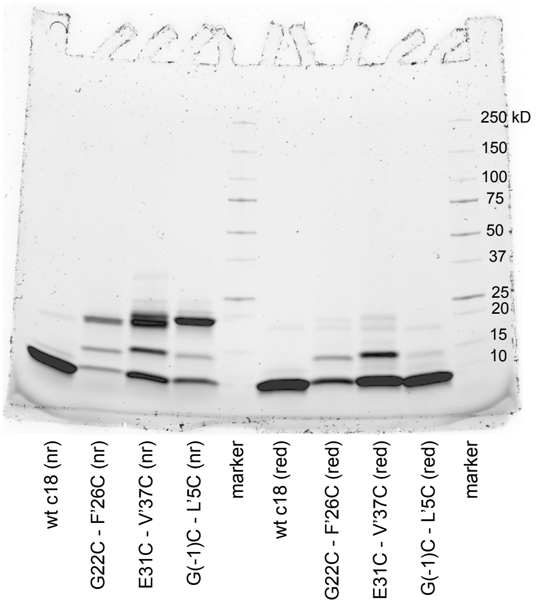

SI Fig. 4: SDS-PAGE of wildtype collagen XVIII trimerization domain and inter-chain disulfide mutants. Samples run on a 4-20% SDS-PAGE stain-free gel (BioRad), left-to-right: non-reduced (1) wt domain, (2) G22C–F'26C, (3) E31C–V'37C, (4) G(-1)C–L'5C; molecular weight marker (Precision Plus Unstained Protein ladder, BioRad); reduced (5) wt domain, (6) G22C–F'26C, (7) E31C–V'37C, (8) G(-1)C–L'5C; marker.

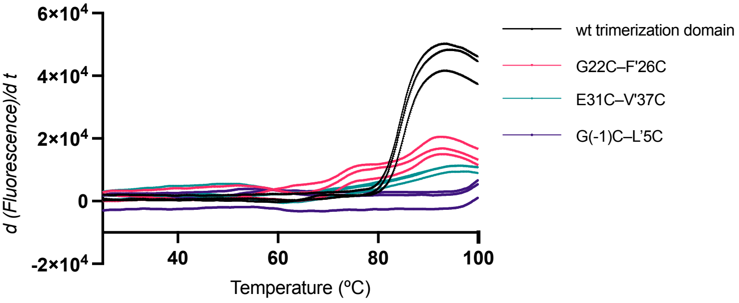

SI Fig. 5: Thermal stability of wildtype collagen XVIII trimerization domain and inter-chain disulfide mutants. Raw DSF melting curves for collagen XVIII trimerization domain wt (black), G22C–F'26C (magenta), E31C–V'37C (teal), and G(-1)C–L'5C (purple), $n = 2$.

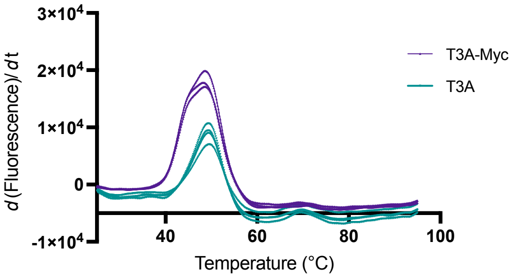

SI Fig. 6: Thermal stability of T3A with and without Myc tag. DSF melting curves for T3A with and without Myc tag in purple and teal, respectively, $n = 2$ and $n = 1$, respectively.

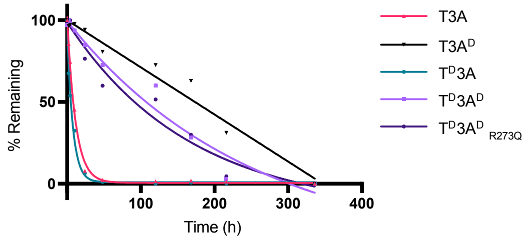

SI Fig. 7: Stability of T3A constructs in rat serum. Unmutated T3A (magenta), T3AD (black), TD3A (teal), TD3AD (light purple), and TD3ADR273Q (dark purple) were incubated in rat serum at 37 °C. Over 336 hours, 5 µL aliquots of each sample were frozen and stored at -80 ºC. Samples were tested via ELISA, using a SARS-CoV-2 spike HexaPro protein-coated plate and probed with an HRP-conjugated anti-Myc mAb to determine binding to the spike protein, $n = 2$. Trendline fit to a single phase decay.
