# Supplementary material for: Development of an ultrahigh affinity, trimeric ACE2 biologic as a universal SARS-CoV-2 antagonist
Source: Commun Biol. 2025 Oct 6;8:1428. doi: 10.1038/s42003-025-08819-w (PMC12501226; doi:10.1038/s42003-025-08819-w)
Supplement: Supplementary file 3 — Description of Additional Supplementary Files [file 42003_2025_8819_MOESM3_ESM.docx]

Description of Additional Supplementary Files

**File name:** Supplementary Data 1

**Description:** Supporting data for SPR experiments in Figure 1b/c.

**File name:** Supplementary Data 2

**Description:** Supporting data for psuedovirus assay experiments in Figure 1d/e.

**File name:** Supplementary Data 3

**Description:** Supporting data for DSF, activity assay, rat serum stability, and MD simulation experiments in Figure 3.

**File name:** Supplementary Data 4

**Description:** Supporting data for chromatograms, DSF, and rat serum stability experiments in SI Figures.

**File name:** Supplementary Data 5

**Description:** Structure factors (.cif format) for 9BNB diffraction data.

**File name:** Supplementary Data 6

**Description:** Structure factors (.cif format) for 9BNC diffraction data.
